# Nutritional problems in children with neuromotor disabilities: an Italian case series

**DOI:** 10.1186/1824-7288-40-61

**Published:** 2014-07-08

**Authors:** Maria Sangermano, Roberta D’Aniello, Grazia Massa, Raffaele Albano, Pasquale Pisano, Mauro Budetta, Goffredo Scuccimarra, Enrico Papa, Giangennaro Coppola, Pietro Vajro

**Affiliations:** 1 Chair of Pediatrics, Department of Medicine and Surgery, Pediatrics Section, University of Salerno, Via Allende, 84081 Baronissi- Salerno, Italy; 2 Pediatrics, AOU Ruggi D’Aragona, Salerno, Italy; 3 Istituto “Antoniano”, Ercolano-Napoli, Italy; 4 Centro “Don Orione”, Ercolano-Napoli, Italy; 5 Department of Medicine and Surgery, Neuropediatrics Section, University of Salerno, Baronissi (Salerno), Italy; 6 ELFID, European Laboratory for Food Induced Diseases, Naples, Italy

**Keywords:** Children, Cerebral palsy, Psychomotor developmental delay, Feeding, Malnutrition, Growth

## Abstract

**Background and aims:**

Several neuromotor disorders share exclusive, although often overlooked, nutritional problems. The objective of this study is therefore to delineate the frequency of malnutrition, evaluate the effectiveness of nutritional care, and identify issues needing to be possibly strengthened when caring for these patients into a general pediatrics department.

**Patients and methods:**

The study included 30 patients, 21 males and 9 females, aged between 2 and 15 years, affected by cerebral palsy, epileptic encephalopathy, and severe psychomotor developmental delay.

Nutritional status was assessed by a dietary questionnaire administered to parents to investigate feeding difficulties; 3 days food diary to quantify daily calorie intake; anthropometrical (weight, height/length, body mass index percentiles, plicometry, specific body segments measurement) and blood (blood count, serum iron, albumin, transferrin, calcium, phosphorus) parameters.

**Results:**

More than 44% individuals of the study population was at risk of malnutrition, according to feeding difficulties, progressive depletion of weight, reduced daily calorie intake, reduced albumin and transferrin levels. This occurred despite a massive caregivers commitment, as documented by almost universal parental constant assistance during their long-duration meals.

**Conclusions:**

Our results individuate the nutritional aspect being still a problem in the care of children with severe neuromotor disability.

## Introduction

Severe neuromotor disabilities are frequently complicated by nutritional problems in children and adolescents, depending on the severity of the underlying disease [1,2].

Feeding is classically the most serious component of the assistance that involves these patients' families. When compromised, after disability, it is a predictor of early death in individuals with cerebral palsy [2]. In fact, oral motor dysfunction, gastro-oesophageal reflux and food refusal reduce intake of nutrients necessary to meet their nutritional needs [3].

Caloric intake of children with cerebral palsy - despite caregiver’s intense efforts - is therefore reported as inadequate [4]. Indeed, malnutrition (especially protein), endocrine dysfunction and neurological impairment act synergistically determining a slowing of linear growth and weight gain, so that standards of growth, height and weight for age in neurologically impaired children are often lower than those of the control population of healthy children [5,6].

Nutritional interventions as a part of an integrated treatment plan should be developed by a multidisciplinary team to improve both patients’ and their families’ quality of life [7]. In countries with a distressed economic system, controversial aspects still remain. These may include lack of too expensive encoded assistance protocols, scarceness of dedicated facilities, or barely available multidisciplinary approach and specialized skills [8].

The objective of this study is therefore to delineate the frequency of malnutrition and to identify issues needing to be possibly strengthened in an Italian general pediatric asset caring also for children with neuromotor disabilities.

### Methods subjects

The study included 30 patients with psychomotor developmental delay (21 males and 9 females), aged between 2 and 15 years, living either at home or part-time at rehabilitation centers. All patients presented a neuromotor damage that could compromise the ability of self-feeding. The diagnoses are shown in Table 1.

**Table 1:** Diagnosis of 30 patients studied

**Disease**	**Number of cases**	**%**
Cerebral palsy	16	53.3
Epileptic encephalopathy	5	16.6
Severe psycomotor developmental delay	5	16.6
Others	4	13.3
Total	30	100

Nutritional aspects were characterized by anamnestic investigation, clinical and anthropometric evaluation. Medical history included a recall of underlying disease, use of chronically medications, number of hospitalizations, occurrence of respiratory diseases, presence of vomiting and regurgitation, frequency of bowel movements. A specific dietary questionnaire (Table online only) was administered to patients’ families to assess the current feeding supply mode (patient posture, caregiver position, tools used, preferred food temperature and texture, occurrence of cough after fluid intake). Calories daily intake was estimated after appropriate instructions through a 3-day food diary filled in by caregivers [9].

To obtain reliable measurements of height and length, specific body segments were measured according to the criteria of Stevenson [10,11]. Data corresponding to the average of two successive measurements of the upper arm and tibia lengths and knee height were inserted into specific equations to obtain an estimate of stature. Skinfold thickness was measured as well to estimate nutritional status.

Blood indicators of malnutrition included the following routine blood chemistry investigations: Red Blood Cell Count, Hemoglobin, hematocrit, Mean Corpuscolar Volume, Mean Corpuscolar Hemoglobin, Mean Corpuscolar Hemoglobin Concentration, serum Iron, Transferrin, Albumin; Calcium, Phosphorus. The study was conducted in compliance with the Declaration of Helsinki and after obtaining informed consent from the parents of study participants.

## Results

Duration and modalities of the meal, any episodes of choking and/or noisy breathing, methods of foods preparation, frequency and stools consistency are summarized in Table 2.

**Table 2:** Summary of parents’ responses to dietary questionnaire

**Question**	**Answer (%)**		
Supply mode	Caregiver-sustained spoon (81%)	Self-sustained spoon (7%)	Bottle (12%)
Direction of food introduction	Centrally (62%)	From top to bottom (15%)	From bottom to top (23%)
Meal duration	<30 min (46%)	30-60 min (35%)	60 min (19%)
Need for assistance during the meal	Always (88%)	Often (8%)	Rarely (8%)
Loss of saliva/regurgitation	Yes (58%)	No (42%)	
Type of food preparation	Liquid (35%)	Dense (54%)	None (11%)
Story of respiratory diseases (bronchopneumonia, aspiration pneumonia)	Yes (39%)	No (61%)	
Choking episodes (cough, noisy breathing, loss of food)	>3 (24%)	< 3 (38%)	None (38%)
Frequence of bowel movements	2-3 days (50%)	3-5 days (50%)	5-7 days (8%)
Medically assisted defecation	Yes (73%)	No (27%)	

**Figure 1:**
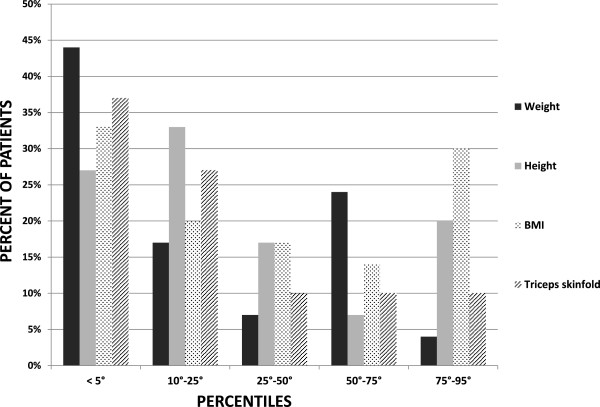
Distribution of growth percentiles for weight, height, BMI and triceps skinfold of the 30 patients included in our study.

Average data (±SD) relative to the current diet composition of subjects included in the study are summarized in Table 3. According to average values of energy needs calculated using Krick’s formula [12], it emerges that patients’ daily calorie intake was particularly low (13/30 patients have a significantly reduced caloric input) with unbalanced macronutrients (especially Carbohydrates and Lipids).As shown in Figure 1, 44%, 33% and 37% of the patients had weight, BMI, triceps skinfold values below the 5th percentile. Four patients (mean age = 8.0 years; range 3–12 years) who had undergone percutaneous endoscopic gastrostomy (PEG) positioning 30 ± 21 DS months before the present survey due to severe malnutrition, reached a BMI between 25th and 50th percentile. Their height however remained in the low range (5th -10th percentile).

**Table 3:** **Calories and diet composition of the 26 boys vs. Italian recommended daily intake levels of energy and nutrients [**[21]**]**

	**Kcal/die**	**Carbohydrates ± SD (%)**	**Lipids ± SD (%)**	**Proteins ± SD (%)**
**Patients**	1092,1 ± 214,8*	134,2 ± 19 (46%)	45,4 ± 8,9 (37%)	45,9 ± 17,7 (17%)
**Reference values**[21]	1474,5 ± 411,17	55-60%	22-30%	12-15%

*Average of energy requirements calculated using Krick’s formula [12].

Laboratory tests results were in the normal reference intervals in most patients. Iron deficiency hypochromic microcytic anemia was found in 4 out of 30 patients. In 12 out of 30 patients serum albumin values were slightly below the lower limits of normal range. Anthropometric measurements along with feeding difficulties emerging from the structured questionnaire identified several patients having a significant weight deficit and increased risk of malnutrition.

## Discussion

This study’s findings appear to be consistent with those reported in the literature, according to which feeding difficulties in children affected by severe neuromotor disabilities impede the daily caloric intake required for their energy needs, resulting in a decrease of linear growth and a serious risk of malnutrition [13,14].

Although the reasons for malnutrition may be different, the supply difficulties of these patients are the prominent ones. Much interestingly, parental questionnaires of our patients show that approximately 90% of subjects required constant assistance during the meal despite an average age of 10 years. The typical duration of meals is between 60 and 120 minutes, and it is often prolonged further by episodes of food regurgitation or suffocation crises, with much energy spent by parents and unquestionable impact on quality of family life. To partly overcome supply difficulties, more than half of those children need meals consisting of thickened food. Caregivers time expenditure was also dedicated to problems related to constipation requiring medical care for obtaining bowel movements in at least 50% of patients [15].

Despite nutritional and dietary measures, daily calorie intake of these children was insufficient, with an impaired macronutrients balance. Consistent with the significant feeding difficulties and insufficient calories intake, regular weight gain was significantly compromised: approximately half of study population has a weight <5th and one third of the population has a BMI <5th percentile.

According to nutritional problems, growth percentiles and results of laboratory investigations, children with severe neuromotor disabilities in the present study are at serious risk of malnutrition.

One of the limits of our study is that the results obtained are based on a small sample of patients with various causes of severe neuromotor impairment (CP, genetic syndromes, epileptic encephalopathies). In the future -with a wider sample- one might think to analyze separately the results of the two groups (i.e. patients with and without cerebral palsy) to get a better insight.

In the case of unsuccessful dietary positional measures, in specific clinical situations a system of alternative nutrition possibly allowing an adequate and balanced caloric intake, may be needed to safeguard patients from the risk of inhalation during the meal and, last but not least, contribute to improve the overall quality of life of both patients and their caregivers as well. The first step in this direction is typically the naso-gastric or, in case of vomiting, the naso-jejunal tube insertion. The disadvantages of the naso-gastric tube are the reduced lumen caliber allowing exclusively the use of liquid formulations, frequent obstruction, possibility of dislocation with risk of aspiration, and the disturbing exterior visibility of the system.

If the prediction of enteral nutrition is more than three months (according to some authors, six months), there is indication for placement of a gastrostomy. In recent years, the endoscopic method has become a safe and rapid execution procedure [16]. Compared to surgical approach, endoscopic procedure does not require general anesthesia but only a short deep sedation and re-feeding can be faster and the duration of hospitalization lower [17]. Benefits brought by PEG on nutritional status of patients with severe neuromotor disability are now well established in the literature [18]. Our study comprised 4 patients (13.3%) who had undergone the positioning of PEG because of nutritional depletion and of a progressive deterioration of the general health status. Although their linear growth remained in the low percentiles, the rest of their anthropometric evaluation and clinical laboratory investigations appeared having benefited from this nutritional intervention. Comparable results reported for the CP children series of the Norwegian Register (PEG in 14% of 131 children completely dependent on assistance during feeding) [13] underscore the necessity of offering these patients a more timely PEG positioning. Due to the relatively low energy expenditure and high body-fat content of children with severe CP, the potential risk of overfeeding with available enteral feeds administered via gastrostomy tube should however be considered and properly balanced [19].

## Conclusions

Our results emphasize the importance of nutritional problems in the management of neurologically compromised children. These problems are often not adequately investigated, and became severe enough to affect child health [20]. Hence the need to include the study and care of nutritional problems in the general assessment of children with severe neuromotor disability, in order to plan appropriate individualized nutritional intervention to prevent serious complication of malnutrition. Besides the mandatory assessment of the weight and the most common laboratory parameters (i.e. blood count, protein electrophoretic profile), it would be appropriate to associate also other techniques for additional more complex assessment of nutritional status (e.g. bioimpedance, indirect calorimetry, assessment of prealbumin, retinol-binding protein, insulin like growth factor, bone density) [21,22].

The complexity of this peculiar nutritional condition (oral-motor incoordination, scoliosis and postures relocation, gastroesophageal reflux, etc.) implies the need of a multidisciplinary interventional approach involving several figures as physical, speech and occupational therapists, orthopedist, physiatrist, dietitian, pediatrician to ensure a more rapid attainment of the objectives and beneficial nutritional properties [7]. Of course this requires knowledge of true incidence of the problem, social implications as well as health care, well encoded protocols, sometimes expensive dedicated facilities, and specialized skills that remain still controversial aspects especially in countries with low or distressed public health-budgets [8].

## Competing interests

The authors declare that they have no competing interests.

## Authors’ contribution

PV and MS designed the study, and prepared the last draft of the manuscript. PV supervised the study. MS coordinated clinical and anthropometric data collection and wrote the first draft. RD made substantial contributions to acquisition and interpretation of data. RA, PP, MB, GS, EP, GC contributed with their own patients, critically improved the study protocol, and revised the manuscript. GM and PP elaborated nutritional data of the patients. All authors read and approved the final manuscript*.*
